# Supporting the Dynamic Careers of Licensed Practical Nurses: A Strategy to Bolster the Long-Term Care Nurse Workforce

**DOI:** 10.1177/15271544211030268

**Published:** 2021-07-07

**Authors:** Cheryl B. Jones, Meriel McCollum, Alberta K. Tran, Mark Toles, George J. Knafl

**Affiliations:** 1 Hillman Scholars Program in Nursing Innovation, University of North Carolina at Chapel Hill, Chapel Hill, NC, United States; 2 Cecil G. Sheps Center for Health Services Research, 2331 University of North Carolina at Chapel Hill, University of North Carolina at Chapel Hill, Chapel Hill, NC, United States; 3 School of Nursing, University of North Carolina at Chapel Hill, Chapel Hill, NC, United States

**Keywords:** long-term care, licensed practical nurses, nursing workforce

## Abstract

As the U.S. population ages and the demand for long-term care increases, an insufficient number of licensed practical nurses (LPNs) is expected in the nursing workforce. Understanding the characteristics of LPN participation in the workforce is essential to address this challenge. Drawing on the theory of boundaryless careers, the authors examined longitudinal employment data from LPNs in North Carolina and described patterns in LPN licensure and career transitions. Two career patterns were identified: (a) the continuous career, in which LPNs were licensed in 75% or more of the years they were eligible to be licensed and (b) the intermittent career, in which lapses in licensure occurred. Findings indicated that LPNs who made job transitions were more likely to demonstrate continuous careers, as were Black LPNs. These findings suggest the importance of organizational support for LPN career transitions and support for diversity in the LPN workforce.

As the U.S. population ages and the demand for long-term care (LTC) increases, an insufficient number of licensed practical nurses (LPNs) is expected in the nursing workforce. Understanding the characteristics of LPN participation in the workforce is essential to address this challenge. Drawing on the theory of boundaryless careers, the authors examined longitudinal employment data from LPNs in North Carolina (NC) and described patterns in LPN licensure and career transitions. Two career patterns were identified: (a) the continuous career, in which LPNs were licensed in 75% or more of the years they were eligible to be licensed and (b) the intermittent career, in which lapses in licensure occurred. Findings indicated that LPNs who made job transitions were more likely to demonstrate continuous careers, as were Black LPNs. These findings suggest the importance of organizational support for LPN career transitions and support for diversity in the LPN workforce.

An aging and growing population, along with expansions in health insurance coverage, has created an increased demand for health care workers in multiple clinical settings across the United States. In particular, long-term care services and supports (LTSS) settings, such as nursing homes, adult day care, assisted living, and home health, have vastly expanded employment options for licensed and unlicensed health care workers. LPNs^1^ are one of two types of licensed nurse that comprise the nursing workforce, along with registered nurses (RNs). LPNs typically work under the supervision of RNs or physicians. They are primarily employed in LTSS settings and are relied on to provide much of the direct care for patients in these settings, alongside nursing support staff such as Certified Nursing Assistants (Coffman et al., 2015; Jones et al., 2018; National Center for Health Workforce Analysis [NCHWA], 2018). LPNs are often viewed as a cost-effective and essential segment of the nursing workforce, especially in LTSS settings that have high patient care needs such as skilled nursing facilities and home health.

Maintaining a sufficient and active supply of LPNs will be critical to address anticipated future demands for the U.S. health care workforce. In 2018, more than 700,000 LPNs were employed in the United States (U.S. Bureau of Labor Statistics, 2018). Demand for both LPNs and RNs in LTSS settings is projected to grow by 46% between 2015 and 2030 (NCHWA, 2018). In addition, by 2030, the Health Resources and Services Administration (HRSA) estimates a projected national shortfall of 151,500 full-time LPNs and a shortage of LPNs across 33 states (NCHWA, 2017b). Thus, an urgent challenge for health care policy makers and administrators is to maintain a sufficient LPN workforce. Two possible approaches for expanding the LPN workforce could be to (a) educate and license more LPNs and (b) increase the participation of inactive LPNs in the workforce, including both licensed and unlicensed LPNs. The first approach is more challenging because of time required to recruit, educate, and license new entrants to the LPN workforce. The latter approach is more appealing in the short run as it provides a more direct and efficient way to add LPNs to the workforce quickly. However, empirical studies are needed to describe LPN participation in the workforce, and particularly their transitions between LTSS and other settings, or into and out of active nursing practice.

In the case of RNs, employment patterns frequently include transitions between part-time and full-time employment, within and between health care organizations, and into and out of the nursing workforce (Gilmartin, 2012). However, patterns of LPN employment and how these patterns compare to known patterns of RNs have not been described. Addressing this gap in research, we analyzed LPN workforce data from 2004 to 2013 to describe the career patterns and participation of LPNs in the workforce and to identify predictors of LPN participation in the workforce. Understanding the career patterns and transitions of LPNs will inform the development of health care system and workforce policies to bolster the largest segment of the LTSS workforce and ensure adequate LPN support and staffing for an aging U.S. population.

## Background

Hall (1996) predicted that careers in the 21st century would be characterized by change that was initiated by employees, not employers, and in response to changes in the lives of employees and their environment. Hall suggested that individual workers would “reinvent” themselves from time to time, changing employers and careers to meet their personal needs, drives, and desires. Hall called this type of career “protean,” after the Greek god Proteus, who could change his shape at will. This type of career is in stark contrast to more traditional conceptualizations of careers, in which an employee joins an organization, stays in, moves up the organizational ladder to higher level positions, and then, after years of service with an employer, retires at the end of their career.

In recent decades, the nature of careers has changed as employees have gained greater ability to move between organizations, vocations, and modes of employment, such as full-time, part-time, per diem, at-home, web-based, and self-employment. Traditional models of work are being replaced by dynamic patterns of employment that are based on the “boundaryless” career, in which employees are more mobile and able to work across organizational and vocational boundaries (Arthur & Rousseau, 1996). Boundaryless careers are characterized by the movement of individuals within and across multiple organizations as they seek out new career opportunities that extend beyond the “boundaries” imposed by a single organization or employment setting (Arthur & Rousseau, 1996; Defillippi & Arthur, 1994). The boundaryless career model allows employees to move through careers more dynamically than up the traditional and hierarchical structural “corporate ladder.” This change in career mobility allows individuals to move through their careers in a “protean” manner, making changes based on personal motivations such as job satisfaction or their own self-evaluation and desires, rather than based on obtaining salary increases or role promotions, and typically within a single organization, alone (Stumpf, 2014).

Alternative career paths in which individuals change their work hours, employment setting, career specialty, employer, and even occupation represent the flexible nature of 21st century career pathways and work structures. However, the flexibility of career paths for LPNs is unknown. LPNs represent a unique sample of workers: They are licensed, skilled, and educated workers, beyond entry-level health care workers, but they do not possess the same license, scope of practice, education, training, and pay of RNs. Furthermore, important demographic and socioeconomic differences exist between LPNs and RNs. While both LPNs and RNs tend to be mostly White, LPNs do represent a more diverse sector of the nursing workforce, with consistently larger proportions of LPNs identifying as Hispanic, Black, and other ethnic and racial backgrounds than RNs (Jones et al., 2018; NCHWA, 2017a). These distinctions between RNs and LPNs signify potential contrasts between the career patterns and behaviors of the two groups, and the ability of each group to make career transitions based on personal, professional, social, and economic benefits and preferences.

Members of underrepresented groups have decreased access to social, economic, psychosocial, and organizational job resources (Schmitz et al., 2019). The diversity within the LPN profession suggests there are structural factors and differences that may differentiate RN career pathways and transitions from those of LPNs. The transition of LPNs to become RNs is a desirable career pathway, as it would bring needed diversity to the RN workforce and further expand and build upon the LPNs’ skillsets; however, there is a gap in knowledge about how often these transitions occur within the LPN workforce. Thus, an examination of the career trajectories of LPNs, separately from those of RNs, is needed to understand the differences between RN and LPN job behaviors and patterns.

## Methods

A retrospective, secondary design was used to examine LPN career patterns and employment behaviors between 2004 and 2013. Data used in this study were obtained from the NC Health Professions Data System, a rich source of data on a variety of licensed health professions in NC. These data were derived from annual licensure files submitted to the Health Professions Data System by the NC Board of Nursing. Licensure files are updated every 2 years as LPN licensure renewals occur, with half of the LPN workforce renewing their licensure each year. At the time of renewal, individual LPNs update their educational, professional, and employment data via an online licensure renewal survey. These data from actively licensed LPNs working in NC between 2004 and 2013 were used to capture transitions that occurred over the 10-year study period.^2^

### Measures

The dependent variable of our analyses was the occurrence of continuous LPN workforce participation between 2004 and 2013. Participation in each of these observed years could have one of three levels: not eligible for licensure (i.e., before the first year of possible LPN licensure), eligible but not licensed, or eligible and licensed (i.e., actively licensed). Continuous workforce participation was determined based on an approximate median split of the number of years that LPNs were observed to be actively licensed in the NC workforce, relative to the number of years that they could have possibly been actively licensed. LPNs who advanced to become RNs remained in the LPN licensure data because they retained their LPN license and could have been employed in either an LPN or RN role. However, for the purposes of this study, we considered these cases as continuous participation in the nursing workforce. Therefore, “continuous workforce participation” was operationalized as the maintenance, or renewal, of an LPN or RN license for 75% or more of possible LPN licensure years (based on the year the LPN earned the degree that qualified them for LPN licensure) during the 10-year study period.

Sociodemographic variables may also influence vocational behavior over the course of a nursing career (Ng et al., 2007). Several variables were included in our analysis to help explain career transitions. First, variables were included that do and do not vary with time. Time-invariant predictors included sociodemographic variables (e.g., gender, race, birth year) and professional variables (e.g., year of LPN licensure, age at LPN licensure, the number of years since licensure, and whether the degree qualifying for LPN licensure was from a U.S.-based LPN program). Several time-varying predictors were included: (a) type of education (i.e., LPNs’ highest degree earned throughout the study period: associate degree^3^, Bachelor of Science in Nursing, Master of Science in Nursing, and doctorate in nursing); (b) the specialty area in which LPNs worked (community-based practice, geriatrics, medical/surgical, pediatrics, and other); (c) their work setting (hospital inpatient, LTC, solo/group practice or hospital outpatient, and other); (d) their employment status (full-time or part-time); and (e) their work location (rural or urban), based on the federal information processing standards code representing the LPN’s address of employment and definitions provided by the Federal Office of Management and Budget Core-Based Statistical Areas.^4^ These categorizations have been described in prior analyses of this LPN licensure data set (Jones et al., 2018).

Time-varying predictors were based on the number of LPN transitions that occurred between 2004 and 2013. Because LPNs could have made more than one transition for any time-varying predictor, transitions were categorized based on the most recent transition made. For example, work location transitions were categorized as “always rural,” “always urban,” “last transition from rural to urban,” or “last transition from urban to rural.” LPNs who made multiple transitions between rural and urban locations during the study period were thus categorized based on their last reported transition.

Missing values were addressed so that the full data set could be used in our analyses. For time-invariant predictors, a missing value was treated as one possible value; for example, possible values for gender were male, female, and missing. For time-varying predictors, only nonmissing values were considered for assessing transitions unless the covariate was always missing during the entire period of an LPN's workforce participation. For example, transitions in employment status were categorized as always missing, always full-time, always part-time, last transition full-time to part-time, or last transition part-time to full-time. Note that the categories other than “always missing” could include years in which employment status was missing; however, in all cases, an employment status value was reported in at least 1 year.

### Analyses

The occurrence of continuous LPN workforce participation was analyzed using logistic regression models. Predictors could, in some cases, take on many different values, especially if a predictor was time-varying. For example, work setting, a time-varying predictor, had four nonmissing response options (hospital inpatient, LTC, either solo/group practice or hospital outpatient, and other) or could also be missing. The associated predictor thus had 17 possible levels: always missing, always one of the four possible nonmissing covariate levels, or one of 12 possible last transitions from one response option to another.

To address the wide variability of these patterns, predictors were adjusted one at a time to parsimonious versions, based on grouped sets of predictor levels using multiple adaptive regression splines (MARS) modeling (Friedman, 1991). MARS modeling handles both continuous and categorical predictors, but the analysis only involved categorical predictors. This approach merges all of the possible levels of a categorical predictor into subsets of those levels for which the mean outcome is reasonably treated to be the same within the merged subsets and reasonably treated to be different for different subsets.

MARS modeling was applied to each categorical predictor one at a time. The generated parsimonious categorizations for all the predictors were then combined into a composite multiple logistic regression model to assess joint effects to parsimonious categorizations on the occurrence of continuous LPN workforce participation. Wald χ^2^ tests of effects of each parsimonious categorization were generated adjusting for all the other categorizations. Separate tests of the effect of each level of a categorization compared to a reference level were not generated. All analyses were conducted using SAS® Version 9.4 (SAS Institute Inc., 2017).

## Results

The characteristics of the LPNs in the sample are described in Table 1. LPNs were primarily female (94%), White (68.7%), and earned their LPN licensure qualifying degree from a U.S. nursing school (94.5%). A total of 32,279 nurses were observed to be licensed as LPNs at least once during the 2004–2013 time period and more than half (57.7%) of LPNs worked for at least 1 year in LTC. Nearly a third (*n* = 11,484, 35.6%) of LPNs in our sample were licensed in all (100%) of their possible years of licensure (based on the year the LPN earned the degree that qualified them for LPN licensure). A total of 15,842 or 49.1% of the LPNs were licensed in 75% or more of their possible years of licensure; as this was an approximate median split of possible licensure years, we operationalized this as continuous workforce participation.

**Table 1.** Frequencies for LPN Characteristics.

Variable	Definition	*n* (%)^a^
Gender	Missing	2 (0.01)
	Male	1,963 (6.1)
	Female	30,314 (93.9)
Race	Missing	245 (0.8)
	White	22,163 (68.7)
	Black	8,031 (24.9)
	American Indian	479 (1.5)
	Hispanic	457 (1.4)
	Asian	303 (0.9)
	Other	601 (1.9)
Birth year	1946–1955	8,463 (26.2)
	1956–1964	6,617 (20.5)
	1965–1973	7,159 (22.2)
	1974–1991	10,040 (31.1)
First possible year of LPN licensure	1938–1985	8,563 (26.5)
	1986–1995	5,910 (18.3)
	1996–2005	8,877 (27.5)
	2006–2013	8,929 (27.7)
Age in first possible year of LPN licensure	Missing	1 (0.00)
	16–22	7,739 (24.0)
	23–27	7,983 (24.7)
	28–34	8,313 (25.8)
	35–68	8,243 (25.5)
Years of possible LPN licensure	0–4	7,929 (24.6)
	5–12	8,124 (25.2)
	13–27	8,355 (25.9)
	28–66	7,871 (24.4)
Degree qualifying for LPN licensure from a U.S. school	Missing	171 (0.5)
	No	1,603 (5.0)
	Yes	30,505 (94.5)

*Note*. LPN = licensed practical nurse.

^a^Out of 32,279 LPNs.

In this study, we were interested in finding potential patterns of workforce participation among the LPNs. There were 3^10^ or 59,049 possible participation trajectories, or series of transitions that form a trajectory pattern, over the 10-year period. From these, 624 licensure patterns were observed. The most commonly occurring pattern (7,108 or 22.0%) was for LPNs to have qualified for licensure in 2004 and have been licensed in all 10 years of the study period. This pattern and 24 other commonly occurring patterns were observed in about two-thirds (21,544 or 66.8%) of the total LPN sample; for example, the second most commonly occurring pattern was LPNs who were licensed in 2004, and eligible but not licensed from 2005 to 2013 (1,530 or 4.7%). The 25 most commonly occurring trajectories for this LPN sample are shown in Table 2. The remaining third of the total LPN sample (10,735 or 33.2%) were observed to have one of 599 different kinds of licensure patterns.

**Table 2.** Most Commonly Occurring LPN Workforce Licensure Patterns.

Index	Pattern	*n* ^a^	%	Cumulative %
1	Licensed every year 2004–2013	7108	22.0	22.0
2	Licensed 2004, eligible but not licensed 2005–2013	1530	4.7	26.8
3	Licensed 2004 and 2005, eligible but not licensed 2006–2013	1161	3.6	30.4
4	Licensed 2004–2006, eligible but not licensed 2007–2013	1024	3.2	33.5
5	Licensed 2004–2007, eligible but not licensed 2008–2013	892	2.8	36.3
6	Licensed 2004–2008, eligible but not licensed 2009–2013	813	2.5	38.8
7	Licensed 2004–2009, eligible but not licensed 2010–2013	773	2.4	41.2
8	Licensed 2004–2011, eligible but not licensed 2012–2013	684	2.1	43.3
9	Licensed 2004–2010, eligible but not licensed 2011–2013	676	2.1	45.4
10	Ineligible 2004–2011, licensed 2012–2013	671	2.1	47.5
11	Ineligible 2004–2012, licensed 2013	642	2.0	49.5
12	Ineligible 2004–2010, licensed 2011–2013	623	1.9	51.4
13	Licensed 2004–2012, eligible but not licensed 2013	605	1.9	53.3
14	Ineligible 2004–2009, licensed 2010–2013	548	1.7	55.0
15	Ineligible 2004–2007, licensed 2008–2013	483	1.5	56.5
16	Ineligible 2004–2008, licensed 2009–2013	456	1.4	57.9
17	Eligible but not licensed 2004, licensed 2005–2013	404	1.3	59.1
18	Ineligible 2004–2006, licensed 2007–2013	347	1.1	60.2
19	Ineligible 2004–2011, eligible but not licensed 2012, licensed 2013	340	1.1	61.3
20	Ineligible 2004–2005, licensed 2006–2013	331	1.0	62.3
21	Eligible but not licensed 2004–2005, licensed 2006–2013	324	1.0	63.3
22	Eligible but not licensed 2004–2006, licensed 2007–2013	312	1.0	64.3
23	Ineligible 2004, licensed 2005–2013	275	0.9	65.1
24	Eligible but not licensed 2004–2007, licensed 2008–2013	269	0.8	66.0
25	Ineligible 2004–2009, eligible but not licensed 2010, licensed 2011–2013	263	0.8	66.8

*Note*. LPN = licensed practical nurse.

^a^Out of 32,279 LPNs.

LPN employment characteristics are described in Table 3. Overall, these covariates did not change, or changed only one time for a large proportion of the total sample. The most frequent change was a single transition between specialty areas; only 5% (1,565) of the LPNs had more than one change in these covariates.

**Table 3.** Frequencies of Changes in Employment Characteristics During 2004–2013.

Variable	Number of changes	*n* (%)^a^
Highest degree	0	31,573 (97.8)
	1	704 (2.2)
	2	2 (0.01)
Specialty	0	26,099 (80.9)
	1	4,615 (14.3)
	2	1,281 (4.0)
	3	256 (0.8)
	4	27 (0.1)
	5	1 (0.00)
Work setting	0	26,507 (82.1)
	1	4,362 (13.5)
	2	1,169 (3.6)
	3	208 (0.6)
	4	31 (0.1)
	5	2 (0.01)
Employment status	0	28,291 (87.6)
	1	3,115 (9.7)
	2	756 (2.3)
	3	100 (0.3)
	4	16 (0.05)
	5	1 (0.00)
Work location	0	28,780 (89.2)
	1	2,611 (8.1)
	2	767 (2.4)
	3	106 (0.3)
	4	14 (0.04)
	5	1 (0.00)

^a^Out of 32,279 LPNs.

The characteristics of LPNs with continuous and noncontinuous workforce participation are presented in Table 4. These two groups were relatively similar in terms of gender, race, and degree qualifying for licensure from U.S. versus non-U.S.-based programs; however, the continuous workforce participation group had a slightly higher percentage of Black LPNs (26.5% vs. 23.3% in the noncontinuous workforce group) and lower percentages of LPNs born or licensed in earlier years. As expected, there were higher proportions of “always missing” values for both specialty and highest degree transition variables in the noncontinuous workforce participation group.

**Table 4.** Comparison of LPNs With Continuous Versus Noncontinuous Workforce Participation.

Variable	Category	Continuous participation *n* (%)^a^	Noncontinuous participation *n* (%)^b^
Gender	Male	869 (5.5)	1,094 (6.7)
	Female or missing	14,973 (94.5)	15,343 (93.3)
Race	Missing	21 (0.1)	224 (1.4)
	Hispanic	168 (1.1)	289 (1.8)
	White, American Indian, Asian, or other	11,459 (72.3)	12,087 (73.5)
	Black	4,194 (26.5)	3,837 (23.3)
Birth year	1946–1955	3,694 (23.3)	4,769 (29.0)
	1956–1964	8,556 (54.0)	8,643 (52.6)
	1965–1991	3,592 (22.7)	3,025 (18.4)
First possible year of LPN licensure	1986–1995	3,629 (22.9)	5,248 (31.9)
	1996–2005	6,851 (43.2)	7,622 (46.4)
	2006–2013	5,362 (33.8)	3,567 (21.7)
Years of possible LPN licensure	0–4	3,553 (22.4)	4,376 (26.6)
	5–26	8,076 (51.0)	8,403 (51.1)
	27–66	4,213 (26.6)	3,658 (22.3)
Degree qualifying for LPN licensure from a U.S. school	Missing	47 (0.3)	124 (0.8)
	Yes	14,895 (94.0)	15,610 (95.0)
	No	900 (5.7)	703 (4.3)
Transitions in highest degree	Always missing	1,526 (9.6)	5,870 (35.7)
	Always diploma, always BSN, always MSN, always doctorate, or any other last transition	13,727 (86.6)	10,472 (63.7)
	Last transition diploma to AD	589 (3.7)	95 (0.6)
Transitions in specialty	Always missing	1,263 (8.0)	4,303 (26.2)
	Always medical/surgical	452 (2.9)	785 (4.8)
	Always community-based practice, always pediatrics, or always other	5,166 (32.6)	5,312 (32.3)
	Always geriatrics	4,575 (28.9)	4,243 (25.8)
	Any last transition	4,386 (27.7)	1,794 (10.9)

*Note*. LPN = licensed practical nurse; BSN = Bachelor of Science in Nursing; MSN = Master of Science in Nursing; AD = associate degree.

^a^Out of 15,842 LPNs.

^b^Out of 16,437 LPNs.

Table 5 presents the list of individual parsimonious categorizations of predictors of continuous LPN workforce participation. Categories for each predictor were ordered by the increasing chance of continuous LPN workforce participation (e.g., participation in the LPN workforce for 75% or more of their possible years of licensure). Age at first year of possible LPN licensure was not included in Table 5 because the categories for this covariate were merged into one constant category so that it could be considered to have had no effect on continuous workforce participation.

**Table 5.** Parsimonious Individual Categorical Predictors of Continuous NC LPN Workforce Participation During 2004–2013.

Predictor	Categories	*n* (%)^a^	Estimated chance of continuous workforce participation^b^
Gender	Male	1,963 (6.1)	0.443
	Female or missing	30,316 (93.9)	0.494
Race	Missing	245 (0.8)	0.086
	Hispanic	457 (1.4)	0.368
	White, American Indian, Asian, or other	23,546 (72.9)	0.487
	Black	8,031 (24.9)	0.522
Birth year	1946–1955	8,463 (26.2)	0.436
	1956–1964	6,617 (20.5)	0.543
	1965–1991	17,199 (53.3)	0.497
First possible year of LPN licensure	1986–1995	14,473 (44.8)	0.473
	1996–2005	8,877 (27.5)	0.409
	2006–2013	8,929 (27.7)	0.601
Years of possible LPN licensure	0–4	7,929 (24.6)	0.448
	5–26	16,479 (51.1)	0.490
	27–66	7,871 (24.4)	0.535
Degree qualifying	Missing	171 (0.5)	0.275
for LPN licensure	Yes	30,505 (94.5)	0.488
from a U.S. school	No	1,603 (5.0)	0.561
Transitions in highest degree	Always missing	7,396 (22.9)	0.206
	Always diploma, always BSN, always MSN, always doctorate, or any other last transition	24,199 (75.0)	0.567
	Last transition diploma to AD	684 (2.1)	0.861
Transitions in specialty	Always missing	5,566 (17.2)	0.227
	Always medical/surgical	1,237 (3.8)	0.365
	Always community-based practice, always pediatrics, or always other	10,478 (32.5)	0.493
	Always geriatrics	8,818 (27.3)	0.519
	Any last transition	6,180 (19.1)	0.710
Transitions in work setting	Always missing	5,538 (17.2)	0.228
	Always hospital inpatient	2,486 (7.7)	0.365
	Always other	4,831 (15.0)	0.436
	Always long-term care	9,889 (30.6)	0.524
	Always solo/group practice or hospital outpatient	3,763 (11.7)	0.584
	Any other last transition	5,517 (17.1)	0.707
	Last transition hospital inpatient to solo/group practice or hospital outpatient	255 (0.8)	0.803
Transitions in employment status	Always missing	5,543 (17.2)	0.228
	Always part-time	3,438 (10.7)	0.289
	Always full-time	19,310 (59.8)	0.569
	Last transition full-time to part-time	2,267 (7.0)	0.614
	Last transition part-time to full-time	1,721 (5.3)	0.702
Transitions in work locations	Always rural or always urban	28,780 (89.2)	0.466
	Last transition rural to urban or urban to rural	2,499 (10.8)	0.696

*Note*. AD = associate degree; BSN = Bachelor of Science in Nursing; MSN = Master of Science in Nursing; LPN = licensed practical nurse.

^a^Out of 32,279 LPNs.

^b^Estimated probability of having continuous workforce participation based on each predictor separately and not adjusted for the other predictors.

These categorized predictors of Table 5 were combined into a single composite model as joint predictors for continuous LPN workforce participation. Although *p* values for that composite model were not reported in Table 5, all but two of the effects were significant at *p* < .001. Otherwise, degree qualifying for LPN licensure from a U.S. school was significant at 0.004; gender was the only nonsignificant predictor at *p* = .073. These results indicate that the chance of continuous LPN workforce participation depends on all the nongender categorizations of Table 5 after controlling for the other predictors.

In these analyses, as shown in Table 5, we found that continuous workforce participation was predicted by demographic and educational factors, including Black race, birth between 1956 and 1973, first year of LPN licensure in 2006–2013, degree qualifying for licensure from a non-U.S.-based program, and transitions from an LPN diploma to an associate degree. In addition, LPNs who made any career transition, such as transitioning (e.g., from one specialty to another, one work setting to another, between full-time or part-time status, or in rural or urban work location), were also more likely to have continuous workforce participation.

## Discussion

### LPN Career Patterns

In this study, we focused on the LPN workforce in one state, over time, to describe the career transitions and patterns for LPNs, and how LPNs’ personal, professional, and employment characteristics affect their workforce participation. Our research contributes several important findings to the body of literature on LPN career patterns. First, LPNs observed during this study period most commonly made no transitions in their highest degree, specialty area of practice, work setting, employment status, or work location. Compared to LPNs who made no transitions, those LPNs with transitions in their career, such as between full-time to part-time employment, between urban and rural work settings, or between different work settings and/or specialties, were more likely to have continuous careers that were not characterized by lapses in licensure. One explanation for this is that the ability to make job transitions—whether from one job to another, one specialty to the next, or from a rural to an urban setting or vice versa—may be conducive to a worker’s needs for growth, variation, change, and pursuit of better working conditions, pay, or promotional opportunity (Harris et al., 2013). This assumption is consistent with research regarding career advancement within organizations; for example, a study of employees who made vertical transitions within their organization found that making these transitions increased employee engagement and retention (Verbruggen et al., 2015).

Often, the vocational and organizational mobility that is perceived to be beneficial for the individual employee is perceived as a problem for the employer (Feldman & Ng, 2007; Griffeth et al., 2000). Although the pursuit of better working conditions, better wages, better opportunities, or better living situations may be associated with organizational turnover or movement across specialties, this mobility may also keep LPNs actively in practice and working in the nurse workforce. Jones (1996) suggests that within the boundaryless career, workers actively seek out experiences that maintain and expand their skills and increase their attractiveness to potential employers. Likewise, the movement of LPNs across settings, specialties, and organizations may signal a desire to stimulate growth and learning, satisfy basic psychological needs for change (Long & West, 2007), and provide new opportunities for better pay or improved working conditions. The dynamic movement of LPNs that allow them to remain active in the workforce may be motivated by their personal desire to avoid stagnation by pursuing opportunities that maintain or add to their skills, experiences, or “human capital.”

Second, we found that half of the LPNs in our sample were licensed for 75% or more of all years possible during the 10-year period (based on the year when they became eligible for licensure). The remaining half of LPNs, or those with intermittent careers (i.e., LPNs licensed for less than 75% of the years they were eligible to be licensed), represents a segment of the LPN workforce that could reenter active practice and potentially meet the growing demands for LPNs in LTSS and other community-based practice areas. LPNs with intermittent careers experienced “breaks” in licensure, or years in which they could have been but were not, employed and actively practicing as nurses. Although the reasons for these breaks in licensure are unknown, it may be that LPNs were employed in non-LPN jobs during these lapses in LPN employment and that these lapses occurred because of difficulties in finding LPN employment, or other responsibilities, such as family obligations. Additional research is needed to understand the reasons *why* LPNs may interrupt their careers or make job transitions.

Research on LPN turnover behavior also is needed to understand why LPNs make career transitions. Organizations that focus exclusively on increasing productivity and meeting economic or outcomes goals and overlook the career goals of individual employees may be at odds with the needs of LPNs and other bedside patient care providers. For example, organizations with cultures that reflect a commitment to innovation and change, the integration of new ideas, decentralization in decision-making, and encouragement of problem-solving behaviors report lower LPN turnover rates. Similarly, organizations with cultures that are outcomes-focused, focusing largely on achieving quality metrics and productivity goals have higher LPN turnover rates (Banaszak-Holl et al., 2015). Consequently, these employees may choose to leave such organizations or units where there is a mismatch between their own values as caregivers or professionals, and the values or the organization. Identifying and understanding the “push-pull forces” that influence LPN mobility can help identify strategies to address nursing shortages and enhance nurse capacity. The discontinuous nature of the intermittent career path represents potential challenges to maintaining a sufficient workforce supply that meets future demand for LPN services but offers opportunities for organizational, policy, and educational changes that may incentivize and facilitate LPNs’ movements into areas of need.

### Gender and Race

We found that gender was not a significant predictor of continuous LPN workforce participation. This finding is notable when considering wage differences between men and women, and the reasons for these wage differences that are often proposed. Although this study did not examine the wage differentials of male and female LPNs, it is important to note that despite the similar rates of workforce participation, compensation differences between male and female nurses have been reported elsewhere in the literature (Jones & Gates, 2004; Muench et al., 2015; Spetz, 2016; Wilson, 2016). In a survey of RN and LPN compensation, for example, Stokowski et al. (2019) identified a salary gap of $4,000 per year between male and female LPNs. Gender-based wage gaps are often argued to be a consequence of women’s voluntary decreased participation in the workforce due to marriage or raising children (Kahn et al., 2014), or a result of discrimination against women who are perceived to be “less committed” to their jobs owing to their childrearing responsibilities (Budig & England, 2001). Because gender was not a significant predictor of continuous workforce participation, a wage gap between male and female LPNs, especially if it is based on perceived workforce participation, may be unwarranted and should be reevaluated.

We found that Black LPNs were more likely than White LPNs to continuously participate in the LPN workforce. The way that individuals experience their employment and social life is rarely one-dimensional; the social groups to which individuals belong often shape the ways that they are treated and the opportunities that are available to them. If Black LPNs are more likely to continuously participate in the LPN workforce, as we found in our study, then we should see these nurses’ experiences reflected in their lifetime wages and opportunities for promotion or advancement. However, studies instead suggest that Black nurses typically earn less than White and Asian nurses (Moore & Continelli, 2016), have lower job satisfaction compared to White nurses (Xue, 2015), and face more barriers to promotion and advancement than other nurses (Iheduru-Anderson, 2020; Seago & Spetz, 2008). Although studies of wage gaps and worker experiences recognize that differences can arise for a variety of reasons, further research needs to uncover whether mechanisms of discrimination could also exist.

Despite increased attention to diversity and inclusion within education and nursing (Villarruel & Broome, 2020), studies of structural racism within nursing practice and education are urgently needed. Studies of minority students’ or faculty members’ experiences within academia and educational settings often cite issues of structural racism and discrimination (Beard & Julion, 2016; Hassouneh et al., 2012; Whitfield-Harris et al., 2017); however, studies of the experiences of Black and other minority nurses in clinical-based and work practice settings are lacking. Exploring the impacts of race on professional outcomes and opportunities, particularly for LPNs, remains a significant area for workforce studies. Future research should build on these findings and seek to answer additional questions about the effects of continuous workforce participation on LPNs’ career paths and advancement, and the strategies that may need to be employed to further support Black LPNs’ career trajectories.

## Limitations

Our study has several limitations. First, the analyses used in this study were descriptive in nature. It was impossible to determine the reasons for which LPNs make transitions in their careers, or why making transitions would keep LPNs in the workforce longer; future studies should seek to elucidate these findings. Second, the use of secondary data presented issues of omitted variable bias, as these data were collected primarily for administrative and regulatory purposes and not specifically to answer the research question in this analysis. Without data on omitted variables that could affect workforce participation, it is not possible to fully identify the effects of important variables—such as family and job characteristics—on LPNs’ continuous workforce participations. Third, the censoring of these data (e.g., a transition occurring but not observed within the study window) presents additional issues. However, the 10-year study period used in this analysis was, to our knowledge, the longest study period used in analyzing LPN transitions over time.

This study focused on the locations where nurses worked, versus where they lived, yet we acknowledge there may be interactions between nurses’ work and home locations. We chose to focus on the location where nurses worked versus where they lived for several reasons: Work location could have a more immediate influence on nurses’ career change decisions, and, because nurses tend to have many alternate work locations available to them, changing their work location may be an easier and more modifiable factor than changing where they live. We recommend that future researchers consider the choice of work versus living location in future analyses. Finally, this sample was limited to those LPNs who remained licensed in the state of NC throughout the study period. Those LPNs who left NC to practice in another state could not be included in this study, and generalizations made beyond the state should be made with caution.

## Implications for Policy and Practice

Despite the critical role LPNs play in the delivery of LTSS and the increasing demand for these workers, LPN career patterns and behaviors have remained an understudied topic. Characterizing LPN career transitions can help identify strategies to improve LPN workforce participation and develop future policies and health system initiatives that attract LPNs to the workforce, improve LPN workforce capacity, and address health care challenges. Organizations that are able to support LPNs in the transitions that they make are better able to maintain the knowledge and human capital of their LPN workforce, thereby capitalizing on the benefits of their investments. By acknowledging the dynamic careers that LPNs pursue, organizations and policy makers can support these nurses in their transitions by putting in place strategies to retain them, build on their shared nursing knowledge, and ultimately improve the overall capacity of the nursing workforce.

The LPN workforce is a valuable resource for health care, particularly in LTSS settings. With an aging population and an increased demand for these services, there is tremendous potential for improving nurse retention in these areas of high need and improving the U.S. nurse workforce’s capacity. Policy leaders and stakeholders can first work to further understand the motivations of individual LPNs and what they value in their work. Although specific strategies to retain LPNs have not been developed and tested, creating an organizational culture that values employees by emphasizing retention, diversity, and improving the work environment may help improve employee satisfaction and keep LPNs in the workforce. With an increasing organizational and policy focus on increasing the numbers of nurses with Bachelor of Science in Nursing or higher degrees, LPNs may seek opportunities to maximize their extrinsic rewards (e.g., increases in salary, better contracts, or role advancements) in other geographical locations, specialty areas, and settings. Therefore, to retain the LPN workforce, organizations could increase opportunities available to LPNs that support transitions to further develop their education and experiences within their organizations. Rather than seeking to keep LPNs employed and at the same capacity within one particular area, organizational leaders can develop policies and programs that foster and support LPNs as they transition and move across boundaries within the organization, or into new roles.

LPNs bring strong bedside clinical experiences that complement the skills of others on the nursing and health care team. LPN training prepares these nurses with foundational knowledge to be highly adept at performing clinical skills. Opportunities exist therefore for policy makers to build on the individual and shared nursing knowledge of LPNs by clearly defining and establishing their roles as valued members of the care team. For example, across the spectrum of LTSS, LPNs’ knowledge and training prepares them to work alongside other LPNs, RNs, and assistive personnel. Yet, because they are licensed, LPNs also play important roles both in and outside of LTSS, as team members on innovative, integrated models of care that span primary care and physician practices (Bodenheimer, 2007; Fraher, 2016, 2020), and even hospitals. Thus, LPNs bring foundational and shared knowledge, or human capital, that provides flexibility on the care team, enables them to be valuable members of the nursing workforce and health care team, and can support their practice at a level that their regulatory scope allows.

Finally, to build capacity in the overall nursing workforce, policies could be implemented that encourage LPNs who may have left the nursing workforce to return, thereby bolstering the supply of LPNs needed to meet the demand in LTSS, and bringing sorely needed diversity to the overall nursing and health care workforce. This strategy is especially important during times of acute health care workforce shortages. Events such as the COVID-19 pandemic have highlighted the value of quickly mobilizing inactive LPNs and other nurses back into active clinical practice. Although studies examining the successful return of LPNs to the nursing workforce are lacking, findings from studies of RNs who left the workforce offer suggestions for the successful return of nurses, in general. For example, studies have suggested that offering part-time positions, offering flexibility in shift lengths, and offering reentry training for those nurses who have not practiced in several years aid in the successful reentry of RNs into the workforce; these efforts may similarly assist and incentivize LPNs to return to the workforce, as well (Long & West, 2007). To better inform policies and efforts to enhance health care workforce capacity, future research should examine the ways in which nurses, and different subgroups of nurses (e.g., RNs vs. LPNs), mold or adjust their career paths to fit their individual needs and interests and how these career paths contribute to the nursing workforce at large.

## Conclusions

In this study, we found that LPNs with continuous workforce participation exhibited dynamic transition behaviors, transforming their career paths in a number of different ways. Although this study does not provide insights about *why* LPNs made these transitions, we can speculate that individuals were driven to make transitions because it benefitted them in some way. Policies that promote the flexibility of nursing, foster professional growth, and support LPNs in making transitions and moving easily across such organizational and vocational boundaries ultimately may contribute to a more stable LPN workforce and thus, a more stable LTSS workforce.

Findings from our study suggest that organizations could benefit by viewing LPN employment as a series of opportunities that foster and enable them to make transitions throughout their careers. Rather than expecting LPNs to work in one position, organization, or setting throughout their entire careers, organizations and policy leaders should embrace the dynamic patterns of LPN careers and recognize the value of keeping LPNs active in the workforce.

## Notes

1 Also called Licensed Vocational Nurses (LVNs) in some states.

2 Major changes were introduced to LPN licensure renewal surveys distributed after 2013. Therefore, to avoid changes that occurred in the way these data were collected, the latest time period used in this analysis was 2013.

3 Associate’s degrees were not distinguished by category of degree in the data set; thus, an associate degree could be an Associate’s Degree in Nursing (ADN) or an AD in a nonnursing field.

4 We focused our analysis of LPN career change to include the effect of their work location, along with other variables. While we acknowledge a possible interaction between work and living locations and the effects LPN job change, conceptually, we believe LPNs’ work location is likely to have the most relevancy to their decision to make a career change.
